# Supplementary material for: Targeting CCNE1 amplified ovarian and endometrial cancers by combined inhibition of PKMYT1 and ATR
Source: Nat Commun. 2025 Apr 1;16:3112. doi: 10.1038/s41467-025-58183-w (PMC11962063; doi:10.1038/s41467-025-58183-w)
Supplement: Supplementary file 1 — Supplementary Figs. [file 41467_2025_58183_MOESM1_ESM.pdf]

Supplementary Figures and legends

Supplementary Figure 1

Connect With Figure 1

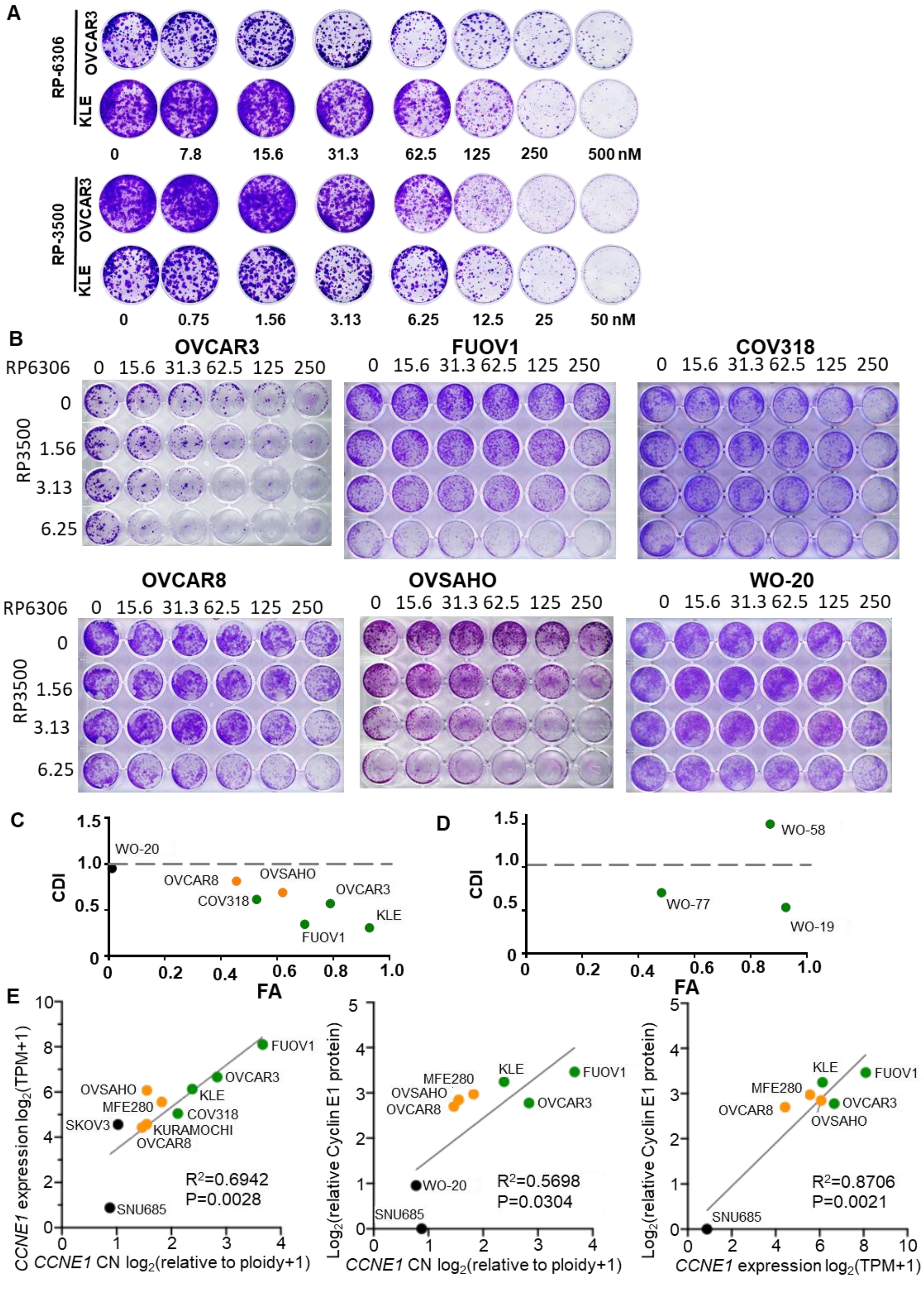

Supplementary Figure 1. Combination of PKMYT1i-ATRi synergistically inhibited viability in cells depending on Cyclin E1 level. (A) Colony formation analysis for RP-6306 and RP-3500 monotherapies in OVCAR3 and KLE. **(B)** Colony formation analysis of HGSOC cells treated with RP-6306, RP-3500 and combinations at indicated dosages for 10 days. (**C**) Coefficient of drug interaction (CDI) and Fraction affected (FA) calculation of colony formation in Figure 1E-F. (**D**) Coefficient of drug interaction (CDI) and Fraction affected (FA) calculation of organoids in Figure 1H. **(E)** Correlation analysis of *CCNE1* expression versus copy number (left), Cyclin E1 protein expression versus *CCNE1* copy number (middle), and Cyclin E1 protein expression versus *CCNE1* expression (right) for the indicated cell lines. TPM: transcripts per million. *CCNE1* copy number and expression data available from DepMap (https://depmap.org/portal; accessed May 28,2024). Cyclin E1 protein expression data available from Xu et al., 2021[1].

Supplementary Figure 2

Connect With Figure 2

A

	IC50 (nM)	
	RP3500	RP6306
SNU685 CCNE1 NO DOX	66.26	794.1
SNU685 CCNE1 +DOX	54.74	165.1

B

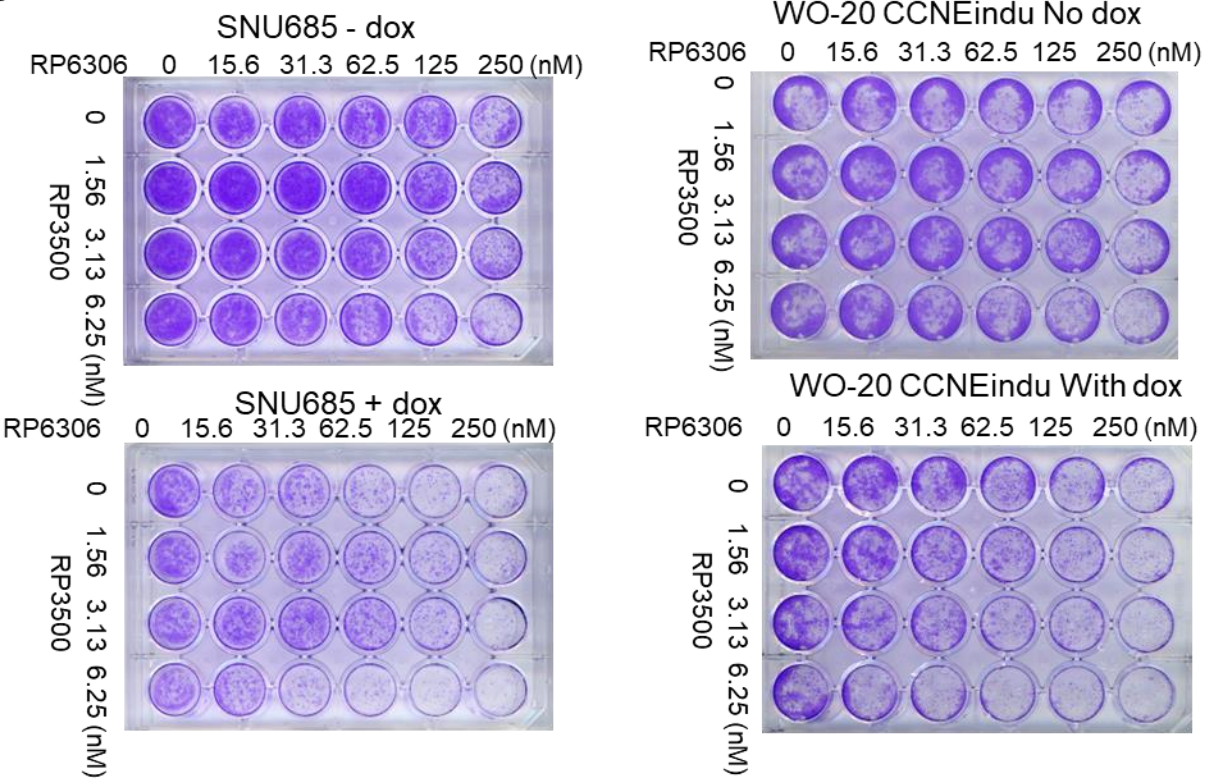

C

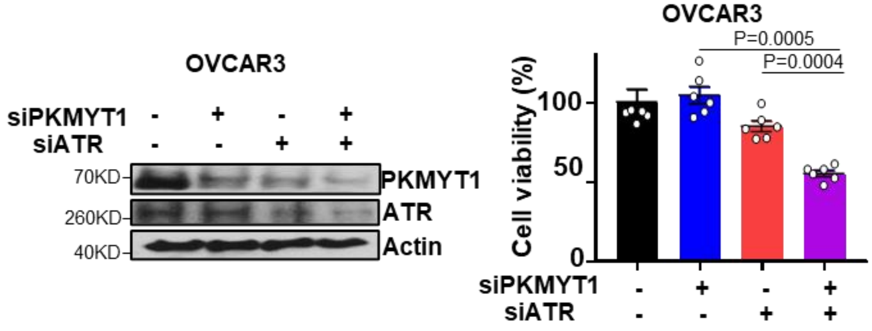

Supplementary Figure 2. Combination of PKMYT1i-ATRi synergistically inhibited cells depending on CCNE1 induction. (A) IC50 of RP3500 and RP6306 in SNU685 *CCNE1*^{inducible} cells with or without Cyclin E1 induction. **(B)** Colony formation analysis of RP6306-RP3500 combination in SNU685 and WO-20 with or without Cyclin E1 induction for 10 days. (C) Measurement of PKMYT1 and ATR protein by western blot after knockdown with siRNAs for 24hrs, and detection of cell viability after siRNA transfection for 48hrs. n=6; Mean + SD. Significance determined by one-way ANOVA followed by Tukey's multiple comparisons test for (C).

Supplementary Figure 3

Connect With Figure 3

A

PDX Model	Cancer Type & Histology	Patient Treatment History	Genetic Mutations*	Copy Number Alterations*
WO-19	High grade serous OVCA	Recurrent platinum resistant, tumor collected at 2'CRS	TP53 c.C1024T (p.R342X)	CCNE1 AMP (CN 11-23)<br>ARAF AMP (CN 11.1)
WO-77	High grade serous OVCA	Recurrent platinum resistant, tumor collected from office vaginal biopsy	TP53 c.G646A (p.V216M)<br>PIK3CA c.A1034T (p.N345I)	CCNE1 AMP (CN 9.1)<br>MYC AMP (CN 10.4)
WU-115	Grade 2 endometrioid EMCA	Newly diagnosed, stage IA	TP53 c.884_909del (p.P295fs)	CCNE1 AMP (CN 35)

*By targeted sequencing

B

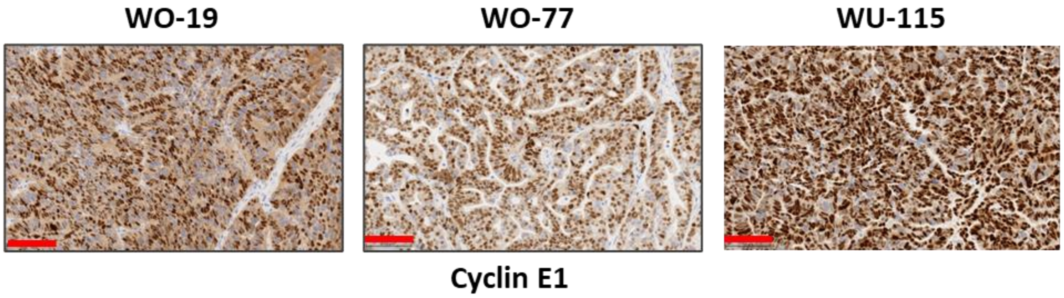

Xenograft model	Animal ID	H-Score	Average H-score
WO-19	51	260	258
	1592	255	
WO-77	3898	230	220
	1890	210	
WU-115	1378	275	280
	1377	285	

Supplementary Figure 3. Characterization of *CCNE1* amplified PDXs. (A) Genetic mutation and amplification information of PDX tumors. **(B)** Measurement of Cyclin E1 protein level in three PDX models by IHC. Scale bar: 100 µm.

Supplementary Figure 4

Connect With Figure 3

A

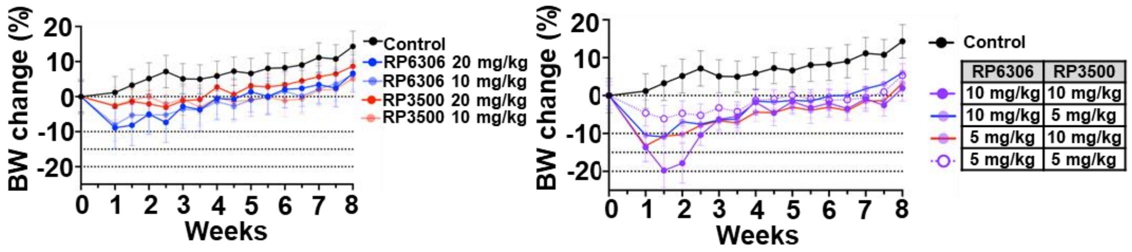

	Con	RP6306 (mg/kg)		RP3500 (mg/kg)		RP6306 + RP3500 (mg/kg)			
		20	10	20	10	10+10	10+5	5+10	5+5
Death	0	0	0	0	0	1	1	0	0
Stop	0	0	0	0	0	3	0	2	0
Level 1 DR	0	0	0	0	0	0	0	0	0
Level 2 DR	0	1	0	0	0	0	2	0	0
Level 3 DR	0	0	0	0	0	1	0	0	0

B

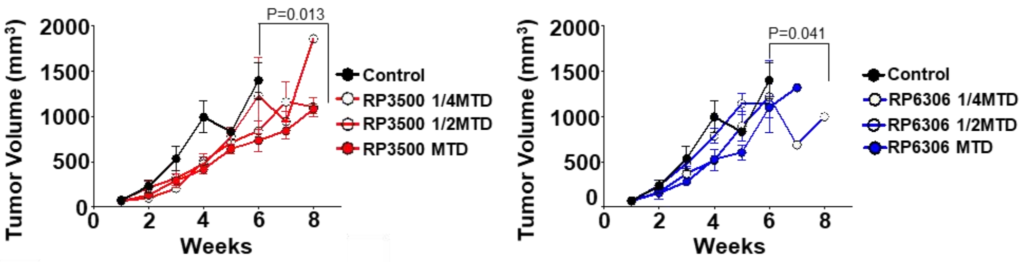

C

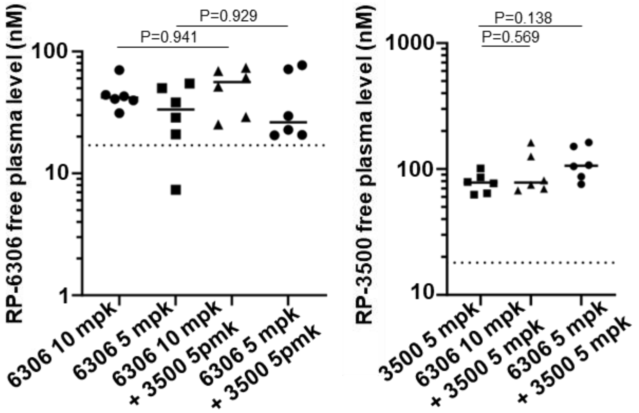

D

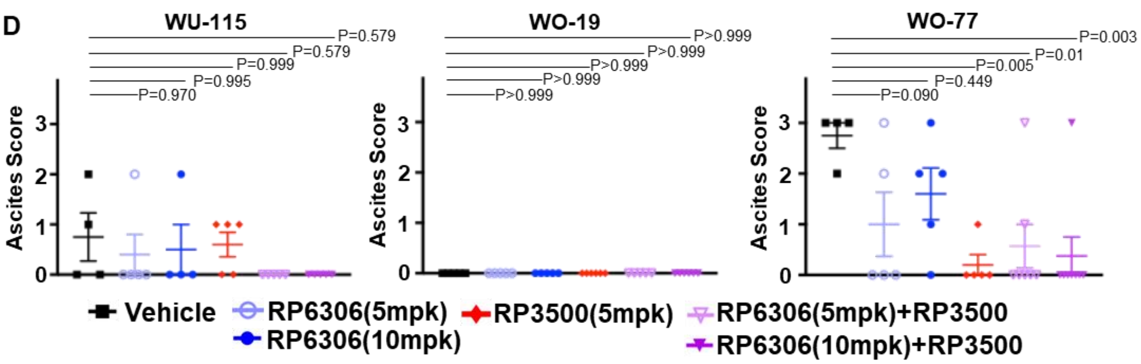

Supplementary Figure 4. PKMYT1i and ATRi shows minimal response even at MTD compared to control. **(A)** PKMYT1i and ATRi monotherapy and Combination PKMYT1i-ATRi is tolerable in NSG mice. Graphs showing percent change in body weight with the indicated treatment after NSG mice were randomized into the indicated groups. Monotherapy arms are shown to the left and combination arms are to the right. n=5; Mean ± SEM. Table (bottom) shows number of mice in each indicated group who required dose reduction or treatment cessation or who died while on treatment. The dose reduction plan was based on percent change in body weight. For a >15% drop, the dose was reduced by 25%. For a >20% drop, the drug was held and restarted at a reduced dose (by 25%) when body weight recovered. **(B)** Tumor volume growth curve for WO-19, *CCNE1* amplified, OVCA PDX model. Mice were randomized into treatment groups as indicated. Treatment continued until disease progression (tumor vol >1000mm^3). n=4 for control, RP-3500 MTD and RP-6306 MTD groups, and n=5 for RP-3500 1/2 MTD, 1/4 MTD and RP-6306 1/2 MTD, 1/4 MTD groups; Mean ± SEM. Longitudinal tumor growth analyzed by Linear Mixed Effects modeling with type II ANOVA and pairwise comparisons across groups. The comparison is between control group and MTD group. **(C)** Pharmacodynamic analysis of RP-6306 (left) and RP-3500 (right) in mice with WO-19 PDX tumors. The mice were treated with indicated dosages of RP-6306 or RP-3500 for 3-4 hrs and collected with blood for analysis. **(D)** Ascites score was analyzed at the end of each PDX experiment for each mouse (0 no ascites, 1 small ascites, 2 medium ascites, 3 large ascites). Significance determined by one-way ANOVA followed by Tukey's multiple comparisons test for (C).

Supplementary Figure 5

Connect With Figure 3

A WU-115 – Tumor Growth

p value	Vehicle	RP6306 5mpk	RP6306 10 mpk	RP3500 5mpk	Both 5-5	Both 10-5
RP6306 5mpk	0.7471		0.1737	0.3878	0.0003	<0.0001
RP6306 10 mpk	0.1867	0.1737		0.5786	0.0240	0.0025
RP3500 5mpk	0.3391	0.3878	0.5786		0.0032	0.0002
Both 5-5	0.0045	0.0003	0.0240	0.0032		0.1935
Both 10-5	0.0008	<0.0001	0.0025	0.0002	0.1935	

WU-115 – Survival

p value	Vehicle	RP6306 5mpk	RP6306 10 mpk	RP3500 5mpk	Both 5-5	Both 10-5
RP6306 5mpk	0.0743		0.3460	0.2941	0.0021	0.0021
RP6306 10 mpk	0.1592	0.3460		0.3967	0.0108	0.0047
RP3500 5mpk	0.1098	0.2941	0.3967		0.0018	0.0018
Both 5-5	0.0027	0.0021	0.0108	0.0018		0.1943
Both 10-5	0.0027	0.0021	0.0047	0.0018	0.1943	

	Vehicle	RP6306 5mpk	RP6306 10 mpk	RP3500 5 mpk	Both 5-5	Both 10-5
Median OS	5.5	9	9.5	10	20	35

B WO-19 – Tumor Growth

p value	Vehicle	RP6306 5mpk	RP6306 10 mpk	RP3500 5mpk	Both 5-5	Both 10-5
RP6306 5mpk	<0.0001		0.0058	0.0012	0.0229	<0.0001
RP6306 10 mpk	0.0796	0.0058		0.9585	<0.0001	<0.0001
RP3500 5mpk	0.0677	0.0012	0.9585		<0.0001	<0.0001
Both 5-5	<0.0001	0.0229	<0.0001	<0.0001		0.0009
Both 10-5	<0.0001	<0.0001	<0.0001	<0.0001	0.0009	

WO-19 – Survival

p value	Vehicle	RP6306 5mpk	RP6306 10 mpk	RP3500 5mpk	Both 5-5	Both 10-5
RP6306 5mpk	0.0254		0.2144	0.6232	0.1598	0.0005
RP6306 10 mpk	0.1166	0.2144		0.2626	0.0173	0.0007
RP3500 5mpk	0.0146	0.6232	0.2626		0.0534	0.0006
Both 5-5	0.0038	0.1598	0.0173	0.0534		0.0082
Both 10-5	0.0007	0.0005	0.0007	0.0006	0.0082	

	Vehicle	RP6306 5mpk	RP6306 10 mpk	RP3500 5 mpk	Both 5-5	Both 10-5
Median OS	2	4.5	3.5	4.5	6.5	9.5

C WO-77 – Tumor Growth

p value	Vehicle	RP6306 5mpk	RP6306 10 mpk	RP3500 5mpk	Both 5-5	Both 10-5
RP6306 5mpk	0.7544		0.0323	0.1101	<0.0001	0.0016
RP6306 10 mpk	0.0139	0.0323		0.5577	0.0244	0.2644
RP3500 5mpk	0.1468	0.1101	0.5577		0.0063	0.0914
Both 5-5	0.0001	<0.0001	0.0244	0.0063		0.1468
Both 10-5	0.0029	0.0016	0.2644	0.0914	0.1468	

WO-77 – Survival

p value	Vehicle	RP6306 5mpk	RP6306 10 mpk	RP3500 5mpk	Both 5-5	Both 10-5
RP6306 5mpk	0.0743		0.0122	0.0235	<0.0001	<0.0001
RP6306 10 mpk	0.1592	0.0122		0.0841	0.0011	0.1261
RP3500 5mpk	0.0840	0.0235	0.0841		0.0003	0.0021
Both 5-5	<0.0001	<0.0001	0.0011	0.0003		0.0449
Both 10-5	0.0002	<0.0001	0.1261	0.0021	0.0449	

	Vehicle	RP6306 5mpk	RP6306 10 mpk	RP3500 5 mpk	Both 5-5	Both 10-5
Median OS	4	3.5	7.5	5.5	12.5	8

D OVCAR3 – Tumor Growth

p value	Vehicle	RP6306 5mpk	RP6306 10 mpk	RP3500 5mpk
RP6306	0.0027		0.0275	<0.0001
RP3500	0.3426	0.0275		<0.0001
Both	<0.0001	<0.0001	<0.0001	

Supplementary Figure 5. Synergy calculation of PKMYT1i-ATRi combination therapies in *CCNE1* amplified WU-115, WO-19, WO-77 PDX models and OVCAR3 model. (A-C) Comparison of tumor growth (Upper panel) and mice survival (Middle panel) between groups were performed in WU-115 (A), WO-19 (B) and WO-77 (C) PDX models. Median overall survival (OS) was calculated for each treatment group (Lower panel). **(D)** Comparison of tumor growth between groups were performed in OVCAR3 model. Longitudinal tumor growth was analyzed by linear mixed effects modeling with type II ANOVA and pairwise comparisons across groups. Data were analyzed for overall survival using Mantel-Cox log-rank test.

Supplementary Figure 6

Connect With Figure 4

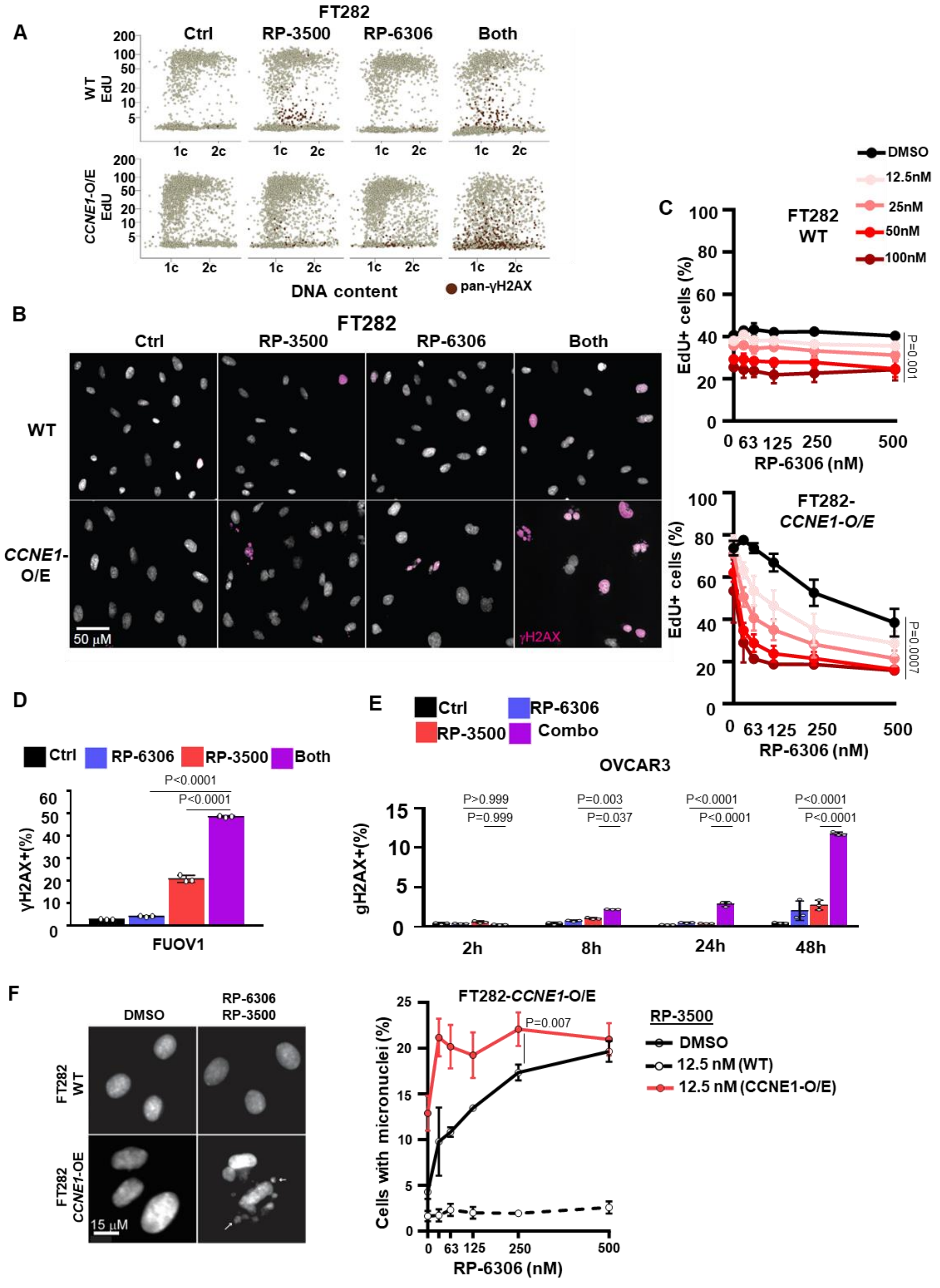

Supplementary Figure 6. Dual inhibition of PKMYT1 and ATR induced DNA damage in *CCNE1* amplified cancers. (A) Representative QIBC plots of γH2AX nuclear intensity, EdU incorporation and DNA content (DAPI) in FT282-hTERT *p53*^{R175H} parental (WT) and *CCNE1*-overexpressing (*CCNE1*-O/E) cells treated with RP-6306 (63 nM), RP-3500 (50 nM) or combination of both treated for 48 h. **(B)** Representative QIBC micrographs of FT282-hTERT *p53*^{R175H} (WT) and *CCNE1*-overexpressing (*CCNE1*-O/E) cells treated either with DMSO, RP-3500 (50 nM), RP-6306 (63 nM) or combination of both. The DAPI (grey) and γH2AX (magenta) channels are merged. **(C)** QIBC quantitation of FT282-hTERT *p53*$^{\mathrm{R175H}}$ parental (upper) and *CCNE1*-overexpressing (lower) EdU^{+} cells in response to the indicated RP-6306/RP-3500 combinations. n=3; Mean + SD. **(D)** FUOV1 cells were treated with RP-6306 (250 nM), RP-3500 (50 nM), or combination of both for 24 h and detected with γH2AX positive cells by flow cytometry. n=3; Mean + SD. (E) Detection of γH2AX positive cells by flow cytometry at low dosage combination of RP-6306 (31.3 nM), RP-3500 (6.25 nM) for indicated time. n=3; Mean + SD. (F) Left, representative micrographs of cells with micronuclei (white arrows) in FT282 parental and *CCNE1*-overexpressing cells following treatment with either DMSO or RP-6306 (31 nM) and RP-3500 (12.5 nM) for 48 h. Right, quantitation of cells with micronuclei in response to the indicated RP-6306/RP-3500 combinations. n=3; Mean + SD. Significance determined by one-way ANOVA followed by Tukey's multiple comparisons test for (C, D), and two-way ANOVA followed by Tukey's multiple comparisons test for (E, F).

Supplementary Figure 7

Connect With Figure 4

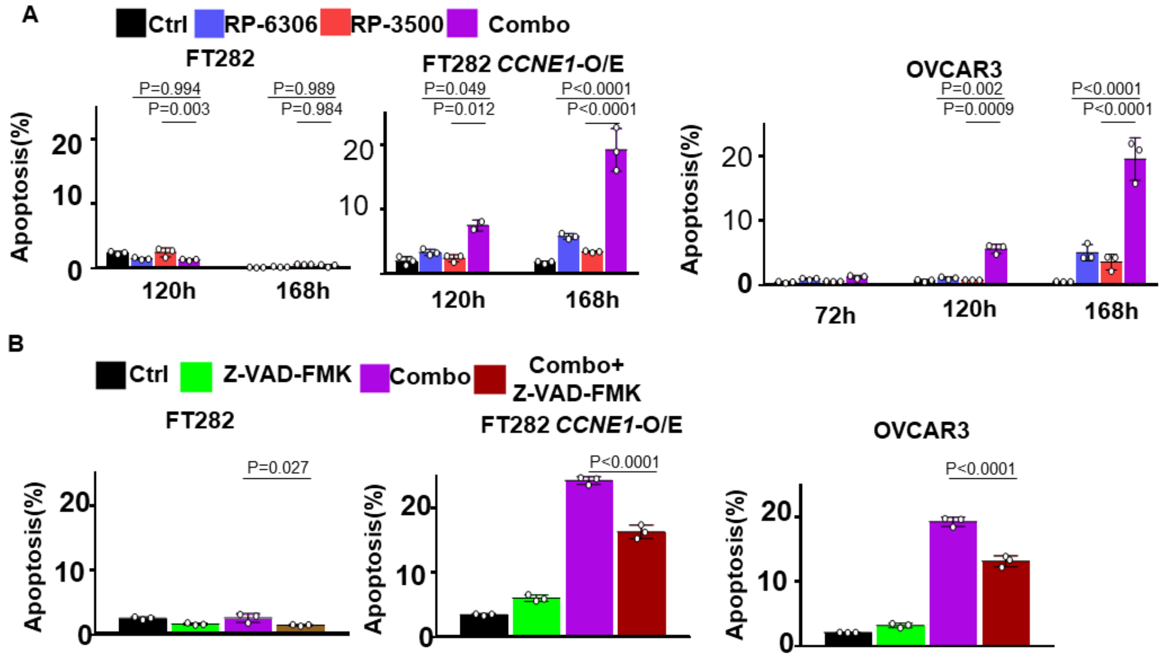

Supplementary Figure 7. Combination inhibition of PKMYT1 and ATR induced cell apoptosis depend in caspases pathway. (A) Detection of cell apoptosis by flow cytometry at low dosage combination of RP-6306 (31.3 nM), RP-3500 (6.25 nM) for indicated hours. (B) Measurement of cell apoptosis with pan-caspase inhibition for 72hrs. Z-VAD-FMK: 20 µM. n=3; Mean + SD. Significance determined by two-way ANOVA followed by Tukey's multiple comparisons test for (A), and one-way ANOVA followed by Tukey's multiple comparisons test for (B).

Supplementary Figure 8

Connect With Figure 5

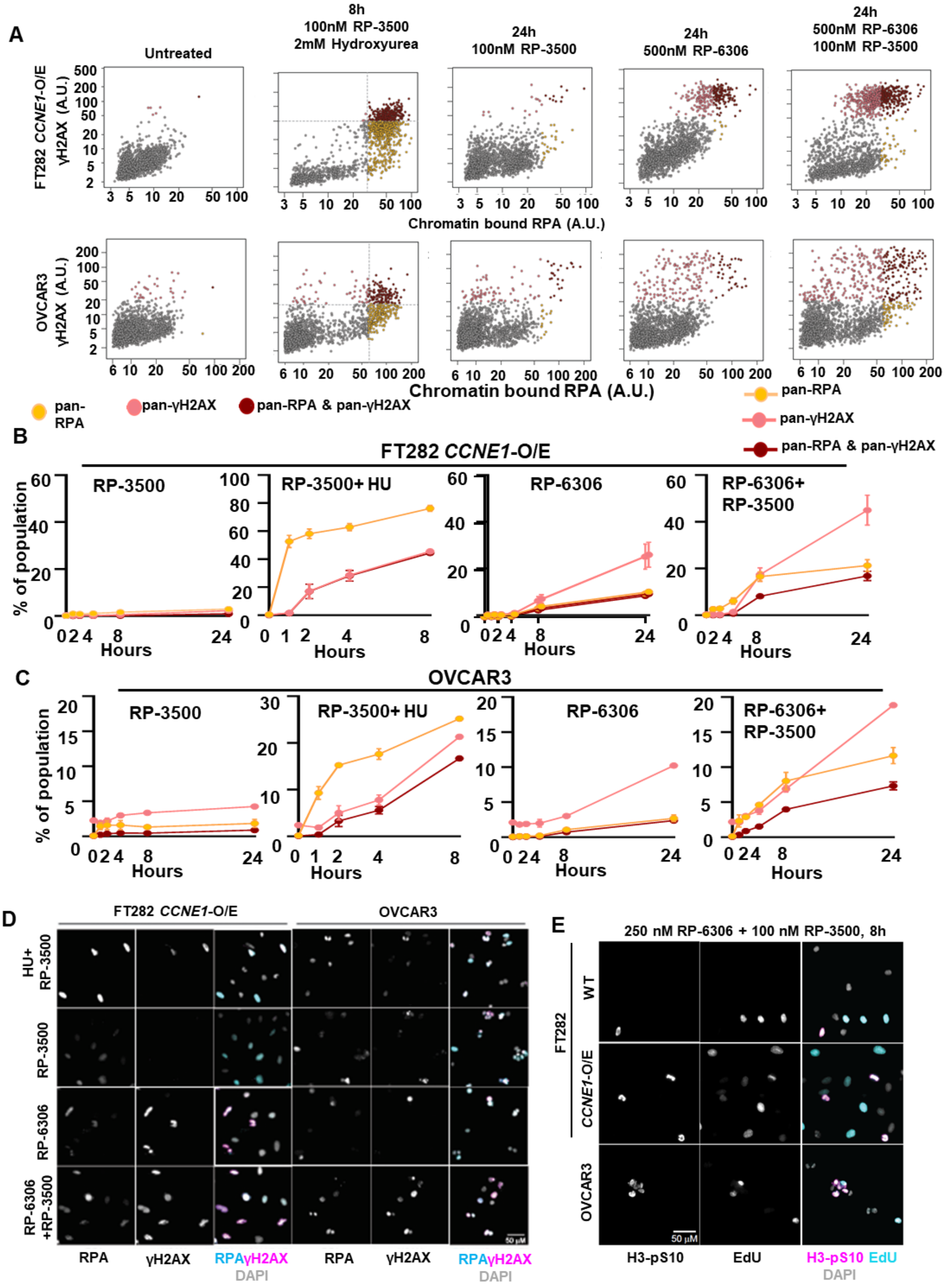

Supplementary Figure 8. Dual inhibition of PKMYT1 and ATR induce premature mitosis in CCNE1 overexpressing cells. (A) Representative γH2AX and RPA nuclear intensity QIBC plots of FT282-hTERT $p53^{R175H}$ *CCNE1*-overexpressing (*CCNE1*-O/E) and OVCAR3 cells treated with the indicated conditions. **(B-C)** QIBC quantitation of FT282-hTERT $p53^{R175H}$ *CCNE1*-overexpressing (*CCNE1*-O/E, B) and OVCAR3 (C) cells with percent of only pan-$\gamma H2AX^{+}$, only pan-RPA^{+} or both pan-$\gamma H2AX^{+}$/pan-RPA^{+} as a function of time after addition of RP-3500 (100 nM), RP-3500 (100 nM) and hydroxyurea (2 mM), RP-6306 (500 nM) or RP-6306 (500 nM) and RP-3500 (100 nM). **(D)** Representative QIBC micrographs of FT282-hTERT $p53^{R175H}$ *CCNE1*-overexpressing (*CCNE1*-O/E) and OVCAR3 cells treated with HU + RP-3500 (2 mM and 100 nM respectively), RP-6306 (500 nM) and RP-3500 (100 nM) and RP-6306 + RP-3500 (500 nM and 100 nM respectively) for 24 h and stained with DAPI (grey), RPA (cyan) and γH2AX antibodies (magenta). The channels are merged and images are representative of three replicates. **(E)** Representative QIBC micrographs of FT282-hTERT $p53^{R175H}$ (WT), *CCNE1*-overexpressing (*CCNE1*-O/E) and OVCAR3 cells treated with RP-6306 (250 nM) and RP-3500 (100 nM) for 8 h and stained with DAPI (grey), EdU (cyan) and a H3-pS10 antibody (magenta). The channels are merged and images are representative of three replicates.

Supplementary Figure 9

Connect With Figure 6

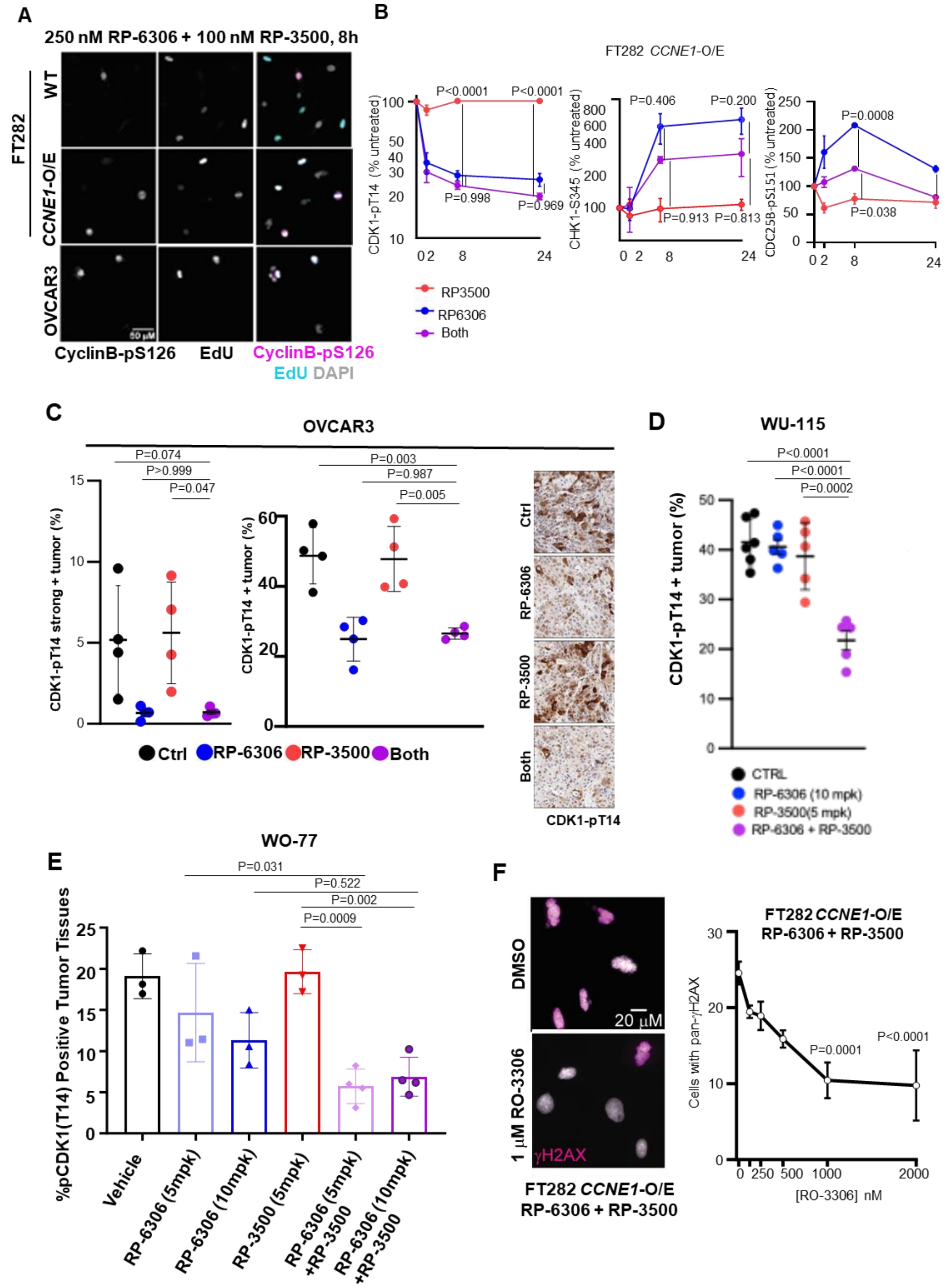

Supplementary Figure 9. Combination of RP-6306 and RP-3500 led to enhanced CDK1 dephosphorylation. (A) Representative QIBC micrographs of FT282-hTERT $p53^{R175H}$ (WT), *CCNE1*-overexpressing (*CCNE1*-O/E) and OVCAR3 cells treated with RP-6306 (250 nM) and RP-3500 (100 nM) for 8 h and stained with DAPI (grey), EdU (cyan) and a cyclin B-pS126 antibody, (magenta). The channels are merged and images are representative of three replicates. **(B)** Quantitation of CDK-pT14 (left), CHK1-pS345 (center) and CDC25B-pS151 (right) as a function of time from whole cell lysates of FT282-hTERT $p53^{R175H}$ *CCNE1*-overexpressing (*CCNE1*-O/E) cells treated with RP-6306 (250nM), RP-3500 (50nM), or both for the indicated times. Each phospho-antibody signal was normalized to the corresponding total protein signal. **(C)** OVCAR3 tumor-bearing mice (C) were administered RP-6306 (5 mg/kg) orally BID, RP-3500 (5 mg/kg) orally QD or combination of both for 3 days, sacrificed 2h post last treatment and tumor tissue was prepared for FFPE. Tumor tissues were stained with CDK1-pT14 antibodies (right) and the percentage of CDK1-pT14 positive tissue (middle) or strong-positive tissue (left) present in the tumor area was quantified by HALO software. n=4; Mean + SD. **(D,E)** Tumor tissue from WO-115 tumor-bearing mice (**D**) administered RP-6306 (10 mg/kg) orally BID, RP-3500 (5 mg/kg) orally QD or combination of both for 10 days, sacrificed 2h post last treatment or WO-77 (E) tumor-bearing mice from figure 3C at end of treatment was prepared for FFPE. Tumor tissues were stained with CDK1-pT14 antibodies and the percentage of CDK1-pT14 positive tissue present in the tumor area was quantified by HALO software. n= 6,5,5,5 (D), . n=3,3,3,3,4,4 (E). **(F)** Representative QIBC micrographs (left) of FT282-hTERT $p53^{R175H}$ *CCNE1*-overexpressing (*CCNE1*-O/E) cells treated with combination of RP-6306 (125 nM), RP-3500 (25 nM) and either DMSO or 1 mM RO-3306 for 24 h and stained with DAPI (grey) and γH2AX antibody, (magenta) and QIBC quantitation of pan-gH2AX staining in FT282-hTERT $p53^{R175H}$ *CCNE1* cells treated with combination of RP-6306 (125 nM) and RP-3500 (25 nM) as a function of CDK1 inhibitor RO-3306 dose. Data are shown as mean + S.D. (n=3)._Significance determined by two-way ANOVA followed by Tukey's multiple comparisons test for B. Significance determined by one-way ANOVA followed by Tukey's multiple comparisons test for C-E. Students' t-test was used to calculate significance in F.

Supplementary Figure 10

Connect With Figure 6

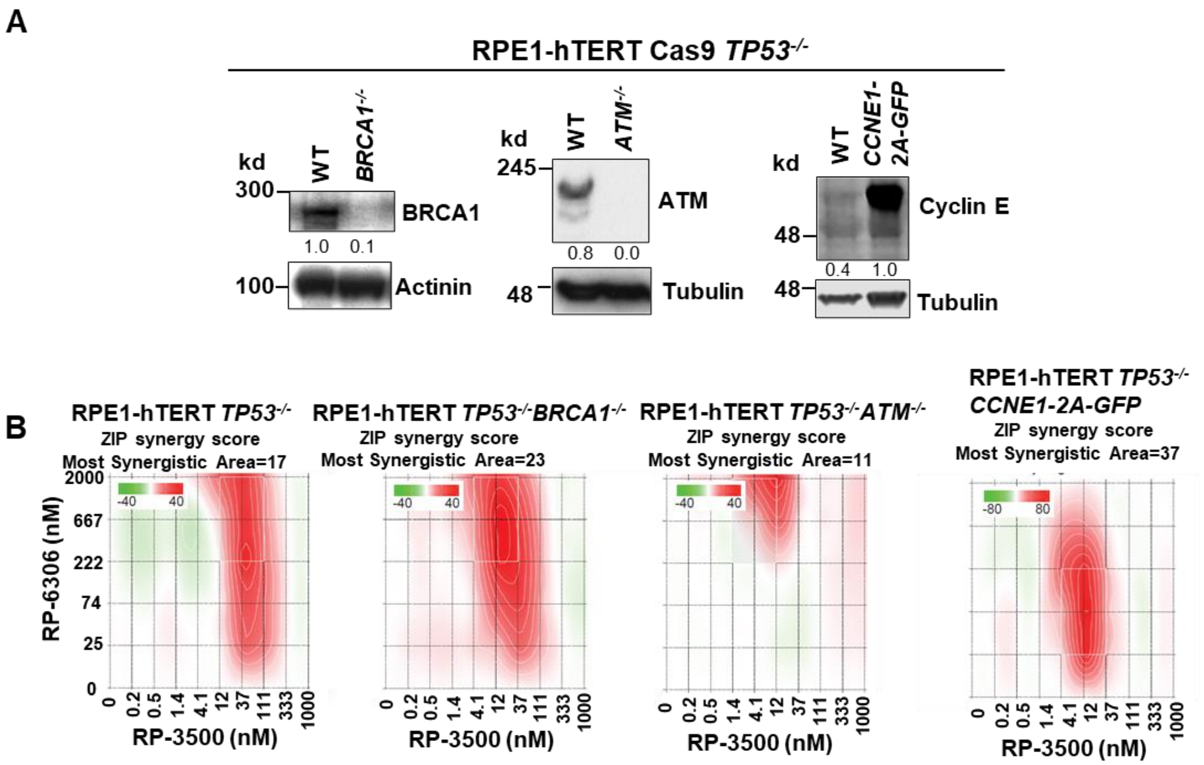

Supplementary Figure 10. (A) Whole cell lysates of RPE1-hTERT *TP53*$^{-/-}$ *ATM*$^{-/-}$ (center) and CCNE1-overexpressing (*CCNE1-2A-GFP,* right) cells were immunoblotted with BRCA1 (left), ATM (center), cyclin E1 (right) and Tubulin (all) specific antibodies. Tubulin is used as a loading control. **(B)** ZIP synergy scores at various dose combinations of RP-6306 and RP-3500 in RPE1-hTERT *TP53*$^{-/-}$ parental, *BRCA1*$^{-/-}$, *ATM*$^{-/-}$ and CCNE1-overexpressing (*CCNE1-2A-GFP*) cells. Score ⩾10 (red color) represents synergy, ⩽-10 (green) represents antagonism. Values were obtained by analyzing mean data from 3 independent biological replicates with SynergyFinder. Significance determined by two-way ANOVA followed by Tukey's multiple comparisons test.

References

1. Xu, H., George, E., Kinose, Y., Kim, H., Shah, J.B., Peake, J.D., Ferman, B., Medvedev, S., Murtha, T., Barger, C.J., et al. (2021). CCNE1 copy number is a biomarker for response to combination WEE1-ATR inhibition in ovarian and endometrial cancer models. Cell Rep Med *2*, 100394. 10.1016/j.xcrm.2021.100394.
