# Supplementary material for: Targeting CCNE1 amplified ovarian and endometrial cancers by combined inhibition of PKMYT1 and ATR
Source: Nat Commun. 2025 Apr 1;16:3112. doi: 10.1038/s41467-025-58183-w (PMC11962063; doi:10.1038/s41467-025-58183-w)
Supplement: Supplementary file 2 — Reporting Summary [file 41467_2025_58183_MOESM2_ESM.pdf]

nature portfolio

Corresponding author(s): Fiona Simpkins, MD

Last updated by author(s): 03/01/2025

Reporting Summary

Nature Portfolio wishes to improve the reproducibility of the work that we publish. This form provides structure for consistency and transparency in reporting. For further information on Nature Portfolio policies, see our Editorial Policies and the Editorial Policy Checklist.

Statistics

For all statistical analyses, confirm that the following items are present in the figure legend, table legend, main text, or Methods section.

n/a	Confirmed	
☐	☒	The exact sample size (*n*) for each experimental group/condition, given as a discrete number and unit of measurement
☐	☒	A statement on whether measurements were taken from distinct samples or whether the same sample was measured repeatedly
☐	☒	The statistical test(s) used AND whether they are one- or two-sided<br>*Only common tests should be described solely by name; describe more complex techniques in the Methods section.*
☐	☒	A description of all covariates tested
☐	☒	A description of any assumptions or corrections, such as tests of normality and adjustment for multiple comparisons
☐	☒	A full description of the statistical parameters including central tendency (e.g. means) or other basic estimates (e.g. regression coefficient) AND variation (e.g. standard deviation) or associated estimates of uncertainty (e.g. confidence intervals)
☐	☒	For null hypothesis testing, the test statistic (e.g. *F*, *t*, *r*) with confidence intervals, effect sizes, degrees of freedom and *P* value noted<br>*Give P values as exact values whenever suitable.*
☒	☐	For Bayesian analysis, information on the choice of priors and Markov chain Monte Carlo settings
☒	☐	For hierarchical and complex designs, identification of the appropriate level for tests and full reporting of outcomes
☒	☐	Estimates of effect sizes (e.g. Cohen's *d*, Pearson's *r*), indicating how they were calculated

Our web collection on statistics for biologists contains articles on many of the points above.

Software and code

Policy information about availability of computer code

Data collection	Microsoft excel, ultrasound measurements by sonosite imager, BioTek Gen5 MTT calorimetric assay software, LSR II, SynergyFinder v2.0 (https://synergyfinder.fimm.fi), Image Studio Lite v5.2.5, Incucyte S3 Software (Sartorius), InCell Analyzer 6000 Acquisition Software v1.0, Aperio AT2 (Leica Biosystems), Studylog v4.4, ZEN 2.3 SP1 (Zeiss) , NIS Elements 5.42.01 (Nikon)
Data analysis	GraphPad Prism (version 7,9), HALO, Flow Jo (version 10), RStudio v1.2.5019, Microsoft Excel 2011, Cellprofiler 3.1.9, Image J (WIN-Java8)

For manuscripts utilizing custom algorithms or software that are central to the research but not yet described in published literature, software must be made available to editors and reviewers. We strongly encourage code deposition in a community repository (e.g. GitHub). See the Nature Portfolio guidelines for submitting code & software for further information.

Data

Policy information about availability of data

All manuscripts must include a data availability statement. This statement should provide the following information, where applicable:

- Accession codes, unique identifiers, or web links for publicly available datasets
- A description of any restrictions on data availability
- For clinical datasets or third party data, please ensure that the statement adheres to our policy

All the data supporting the findings in this work are included in the main article, supplementary information, or source data file. Source data are provided in this paper.

Research involving human participants, their data, or biological material

Policy information about studies with human participants or human data. See also policy information about sex, gender (identity/presentation), and sexual orientation and race, ethnicity and racism.

Reporting on sex and gender	NA
Reporting on race, ethnicity, or other socially relevant groupings	NA
Population characteristics	NA
Recruitment	NA
Ethics oversight	NA

Note that full information on the approval of the study protocol must also be provided in the manuscript.

Field-specific reporting

Please select the one below that is the best fit for your research. If you are not sure, read the appropriate sections before making your selection.

☒ Life sciences ☐ Behavioural & social sciences ☐ Ecological, evolutionary & environmental sciences

For a reference copy of the document with all sections, see nature.com/documents/nr-reporting-summary-flat.pdf

Life sciences study design

All studies must disclose on these points even when the disclosure is negative.

Sample size	The statistical power for animal study is characterize in terms of effect size, defined as A/B, where A = the difference in mean tumor volume at a specific time in control mice compared to each of the treated groups, and B = the estimated pooled variance (in controls and treated mice) for the two groups being compared. An effect size over 1.0 is generally considered to be large, but can be expected to occur in well-controlled studies of treatments with relatively large effects on biological systems. With N = 10 mice per group, the probability is 0.56 of finding a statistically significant difference when treatment induces a difference of 1.0 standard deviation, and the probability is 0.89 of finding a statistically significant difference when treatment induces a difference of 1.5 standard deviations. To ensure statistical power in our results, we transplanted 10 mice per arm. After randomization, the majority of arms had 6 mice per arm with range 5-10.
Data exclusions	For in vitro studies, no data was excluded. For in vivo studies, mice that died for an unknown reason (low tumor burden, stable and normal weights, normal condition scores) would be excluded from analysis.
Replication	All in vitro experiments underwent three biological repeats. All conditions in each experiment had 3-5 replicates. PDX experiments were not repeated using the same model as this would not be acceptable by IACUC.
Randomization	For animal studies, mice were randomized to treatment groups using Tumor Manager randomizing software by Biopticon for PDX studies or using the "stratified" method in Studylogv4.4 software for CDX studies. For QIBC analysis a fixed number of images (24-48) for each condition were collected depending on cell density. Cells were segmented, quantified and the number of cells used for each condition was reduced to match the sample with the lowest cell count. Randomization was conducted using the sample_n method in R.
Blinding	For animal studies, weekly ultrasound measurements, and weight assessments were obtained for treatment groups in a blinded manner. Histology was reviewed by pathologists and image quantification analysis was performed using HALO

Reporting for specific materials, systems and methods

We require information from authors about some types of materials, experimental systems and methods used in many studies. Here, indicate whether each material, system or method listed is relevant to your study. If you are not sure if a list item applies to your research, read the appropriate section before selecting a response.

Materials & experimental systems

n/a	Involved in the study
☐	☒ Antibodies
☐	☒ Eukaryotic cell lines
☒	☐ Palaeontology and archaeology
☐	☒ Animals and other organisms
☒	☐ Clinical data
☒	☐ Dual use research of concern
☒	☐ Plants

Methods

n/a	Involved in the study
☒	☐ ChIP-seq
☐	☒ Flow cytometry
☒	☐ MRI-based neuroimaging

Antibodies

Antibodies used	anti-β-actin (#3700, Cell Signaling Technology, Inc., Danvers, MA),<br>anti-Alpha Actinin (cat.# 05-384, Millipore Sigma, Burlington, MA),<br>anti-CHK1-phosphoS345 (cat. #2348, Cell Signaling Technology Inc., Danvers, MA),<br>anti-CHK1-phosphoS345 (cat#2348, Bethyl Laboratories, Waltham, MA),<br>anti-CHK1 (cat.#2360, Cell Signaling Technology Inc., Danvers, MA),<br>anti-CHK1 (cat.# G-4 sc-8408, Santa Cruz Biotechnology, Dallas, TX),<br>anti-Cleaved caspase-3 (cat.#9664, Cell Signaling Technology Inc., Danvers, MA),<br>anti-γH2AX (cat.#9718, Cell Signaling Technology Inc., Danvers, MA),<br>anti-CDC25B (cat.# TA8-12352, Thermo Fisher Scientific, Waltham, MA),<br>anti-CDC25B-phosphoS151 (cat.# PA5-104568, Thermo Fisher Scientific, Waltham, MA),<br>anti-pCDK1(T14) (cat.# ab58509, Abcam, Cambridge, UK),<br>anti-CDK1(cat.#9116,Cell Signaling Technology Inc., Danvers, MA),<br>anti-cleaved caspase 3(cat.#9664, Cell Signaling Technology Inc., Danvers, MA),<br>anti-Cyclin E1(cat.#4129, Cell Signaling Technology Inc., Danvers, MA),<br>anti-Actin(cat.#3700, Cell Signaling Technology Inc., Danvers, MA),<br>PAX8 (cat. #10336-1-AP, Proteintech, Rosemont, IL),<br>anti-rabbit (cat.#7074, Cell Signaling Technology Inc., Danvers, MA),<br>anti-mouse (cat.# 7076, Cell Signaling Technology Inc., Danvers, MA),<br>anti-mouse Irdye 800CW (cat.# 926-32210, LiCOR Biosciences, Lincoln, NE),<br>anti-mouse Irdye 680CW (cat.# 926-68072, LiCOR Biosciences, Lincoln, NE),<br>anti-rabbit Irdye 800CW (cat.# 925-32213, LiCOR Biosciences, Lincoln, NE) ,<br>anti-rabbit Irdye 680CW (cat.# 926-68073, LiCOR Biosciences, Lincoln, NE) ,<br>anti-pRPA32 (S33, cat#A300-246A, Bethyl Laboratories, Waltham, MA),<br>anti-histone H3 phosphoSer10 (cat# 53348, Cell Signaling Technology Inc., Danvers, MA),<br>Goat anti-Rabbit IgG (H+L), Alexa Fluor 647 (cat# A-21244, ThermoFisher Scientific, Waltham, MA),<br>anti-histone H2A.X (phospho-S139, #05-636, Millipore Sigma,Burlington, MA),<br>anti-RPA32 (Abcam #ab2175, Abcam, Cambridge, UK),<br>anti-Histone H3-phosphoS10 (cat# #9706, Cell Signaling Technology Inc., Danvers, MA),<br>anti-Cyclin B1-phosphoS126 (cat# ab55184, Abcam, Cambridge, UK),<br>anti-ATM (cat#2873, Cell Signaling Technology Inc., Danvers, MA)<br>anti-BRCA1 (Dan Durocher, University of Toronto)<br>anti-CldU antibody (cat#ab6326, Abcam, Cambridge, UK)<br>anti-IdU antibody (cat#347580, BD Pharmigen, Franklin Lakes, NJ)<br>AlexaFluor488 goat anti-mouse IgG (cat# A11029, ThermoFisher Scientific, Waltham, MA),<br>AlexaFluor647 goat anti-rabbit IgG (cat# A21244, ThermoFisher Scientific, Waltham, MA),<br>AlexaFluor555 goat anti-mouse IgG (cat# A28180, ThermoFisher Scientific, Waltham, MA).<br>AlexaFluor 647-conjugated anti-rat IgG secondary antibody (cat# A-21247, ThermoFisher Scientific, Waltham, MA)<br>AlexaFluor 488-conjugated anti-mouse IgG secondary antibody (cat# A-11001, ThermoFisher Scientific, Waltham, MA)
Validation	Cell Signaling Technology antibody undergoes rigorous application-specific validation testing; Analysis of a large panel of cell lines with known target expression levels. Treatment of cells with appropriate kinase-specific activators and/or inhibitors. Correct subcellular localization or treatment-induced translocation. Comparison of results with antibody and isotype control to ensure acceptable signal-to-background ratio. Target-specific signal verified in transfected cells, knockout cells, or siRNA-treated cells. Blocking with antigen peptide to confirm elimination of specific signal. Side-by-side comparison of a new lot with previous lots to ensure lot-to-lot consistency. Bethyl Laboratory has two-phase process ensures that each antibody recognizes distinct epitopes of a target protein. Phase 1: antibody validation process begins with antigen design by selecting between two and four peptides from distinct regions of the target protein. At this stage, valid antibodies must agree in their recognition of the target protein. Agreement between the antibodies is determined in Phase 2. In the second phase of antibody validation process, antibodies were tested for specificity and selectivity using the western blot (WB) method in conjunction with immunoprecipitation (IP).

Eukaryotic cell lines

Policy information about cell lines and Sex and Gender in Research

Cell line source(s)	OVCAR3, FUOV1, KLE cell lines were purchased from ATCC (Manassas, Virginia); FUOV1 was obtained from Leibniz Institute DSMZ; OVCAR8 was obtained from NCI-DTP; Kuramochi, OVSAHO and OVKATE obtained from the Japanese Collection of

	Research Bioresources Cell Bank (JCRB). SNU685 from AcceGen Biotech (Fairview, NJ). FT282-hTERT p53R175H WT (empty vector) and CCNE1 overexpressing cell lines were obtained from Ronny Drapkin in the University of Pennsylvania. The WO-20 primary ovarian cancer tumor cultures were generated in Simpkins laboratory. WO-20 CCNE1 inducible and SNU685 CCNE inducible cells were established by lentivirus stable infection. RPE1-hTERT p53-/- Cas9 BRCA1-/-, ATM-/-, CCNE1-2A-GFP and FT282-hTERT p53R175H WT/CCNE1 overexpressing PCNA-cb-TagRFP cells were obtained from Dan Durocher at the University of Toronto
Authentication	All cell lines authentication was evaluated by Short Tandem Repeat (STR) analysis condcted by Wistar Genomics Core
Mycoplasma contamination	All cell lines tested negative for mycoplasma contamination by PCR analysis by the Cell Center in the University of Pennsylvania or using MycoAlert
Commonly misidentified lines (See ICLAC register)	No cell lines used in this paper are listed in the database of commonly misidentified cell lines (NCBI Biosample)

Animals and other research organisms

Policy information about studies involving animals; ARRIVE guidelines recommended for reporting animal research, and Sex and Gender in Research

Laboratory animals	NSG mice (NOD/SCID IL2Rγ−/−), Female, 8 week old were used for PDX studies and SCID-beige mice, Female, 5-7 weeks old were used for CDX studies
Wild animals	Study did not involve wild animals
Reporting on sex	Female mice were used to generate orthotopic ovarian and endometrial cancer PDX model
Field-collected samples	The study did not involve samples collected from the field
Ethics oversight	For PDX studies Institutional Animal Care and Use Committee and IRB at UPENN provided guidance on the study protocol. For CDX studies animals were housed and experiments were performed at Repare Therapeutics (NEOMED site, Montreal, Canada), which is a CCAC (Canadian Council on Animal Care) accredited vivarium. Studies were conducted under a protocol approved by the NEOMED Institutional Animal Care Committee (NIACC).

Note that full information on the approval of the study protocol must also be provided in the manuscript.

Flow Cytometry

Plots

Confirm that:

☒ The axis labels state the marker and fluorochrome used (e.g. CD4-FITC).

☒ The axis scales are clearly visible. Include numbers along axes only for bottom left plot of group (a 'group' is an analysis of identical markers).

☒ All plots are contour plots with outliers or pseudocolor plots.

☒ A numerical value for number of cells or percentage (with statistics) is provided.

Methodology

Sample preparation	Apoptosis was detected by using an Annexin V Flow Kit (BD Biosciences, Franklin Lakes, NJ). Cells were dissociated with trypsin and labeled with APC-conjugated anti-Annexin V. For Intracellular protein detection, cells membrane were permeabilized and stained with primary antibodies and Alex647 conjugated secondary antibody.
Instrument	BD LSR II, a four laser flow cytometry machine (BD Bioscience)
Software	We used FlowJo version 10 to analyze data.
Cell population abundance	Flow cytometry was used for quantification analysis only, no post sorting fractions were collected
Gating strategy	The cell debris were opt-out based on the FSC/SSC. Only the viable cells were analyzed. Singlet and doublet cells were discriminated using FSC-A/ FSC-W gating. The negative control group was used as a comparison to gate the percentage of positive cells.

☐ Tick this box to confirm that a figure exemplifying the gating strategy is provided in the Supplementary Information.
