# Supplementary material for: Targeting CCNE1 amplified ovarian and endometrial cancers by combined inhibition of PKMYT1 and ATR
Source: Nat Commun. 2025 Apr 1;16:3112. doi: 10.1038/s41467-025-58183-w (PMC11962063; doi:10.1038/s41467-025-58183-w)
Supplement: Supplementary file 3 — Description of Additional Supplementary Files [file 41467_2025_58183_MOESM3_ESM.pdf]

Supplementary Information:

Supplementary Movie 1: *Time-lapse microscopy of WT FT282-hTERT* $TP53^{R175H}$ *cells expressing PCNA-cb-TagRFP.* Cells were imaged every 10 min for 47 h

Supplementary Movie 2: *Time-lapse microscopy of WT FT282-hTERT* $TP53^{R175H}$ *cells expressing PCNA-cb-TagRFP treated with 125 nM RP-6306 + 25 nm RP-3500.* Cells were imaged every 10 min for 47 h

Supplementary Movie 3: *Time-lapse microscopy of WT FT282-hTERT* $TP53^{R175H}$ *CCNE1-OE cells expressing PCNA-cb-TagRFP treated with 125 nM RP-6306 + 25 nm RP-3500.* Cells were imaged every 10 min for 47 h

Supplementary Movie 4: *Time-lapse microscopy of WT FT282-hTERT* $TP53^{R175H}$ *CCNE1-OE cells expressing PCNA-cb-TagRFP treated with 25 nm RP-3500.* Cells were imaged every 10 min for 47 h

Supplementary Movie 5: *Time-lapse microscopy of WT FT282-hTERT* $TP53^{R175H}$ *CCNE1-OE cells expressing PCNA-cb-TagRFP treated with 125 nm RP-6306.* Cells were imaged every 10 min for 47 h

Supplementary Movie 6: *Time-lapse microscopy of WT FT282-hTERT* $TP53^{R175H}$ *CCNE1-OE cells expressing PCNA-cb-TagRFP treated with 125 nM RP-6306 + 25 nm RP-3500.* Cells were imaged every 10 min for 47 h
